# Representation of abstract semantic knowledge in populations of human single neurons in the medial temporal lobe

**DOI:** 10.1371/journal.pbio.3000290

**Published:** 2019-06-03

**Authors:** Thomas P. Reber, Marcel Bausch, Sina Mackay, Jan Boström, Christian E. Elger, Florian Mormann

**Affiliations:** 1 Department of Epileptology, University of Bonn Medical Centre, Bonn, Germany; 2 Faculty of Psychology, Swiss Distance University Institute, Brig, Switzerland; 3 Department of Neurosurgery, University of Bonn Medical Centre, Bonn, Germany; Weizmann Institute of Science, ISRAEL

## Abstract

Sensory experience elicits complex activity patterns throughout the neocortex. Projections from the neocortex converge onto the medial temporal lobe (MTL), in which distributed neocortical firing patterns are distilled into sparse representations. The precise nature of these neuronal representations is still unknown. Here, we show that population activity patterns in the MTL are governed by high levels of semantic abstraction. We recorded human single-unit activity in the MTL (4,917 units, 25 patients) while subjects viewed 100 images grouped into 10 semantic categories of 10 exemplars each. High levels of semantic abstraction were indicated by representational similarity analyses (RSAs) of patterns elicited by individual stimuli. Moreover, pattern classifiers trained to decode semantic categories generalised successfully to unseen exemplars, and classifiers trained to decode exemplar identity more often confused exemplars of the same versus different categories. Semantic abstraction and generalisation may thus be key to efficiently distill the essence of an experience into sparse representations in the human MTL. Although semantic abstraction is efficient and may facilitate generalisation of knowledge to novel situations, it comes at the cost of a loss of detail and may be central to the generation of false memories.

## Abstract 2

Single-neuron representations of stimuli in the human medial temporal lobe at the population level are governed by highly abstract semantic principles, but the attendant efficiency and potential for generalization comes at the cost of confusion between related stimuli.

## Author summary

What is the neuronal code for sensory experience in the human medial temporal lobe (MTL)? Single-cell electrophysiology in the awake human brain during chronic, invasive epilepsy monitoring has previously revealed the existence of so-called concept cells. These cells have been found to increase their firing rate in response to, for example, the famous tennis player ‘Roger Federer’, whether his name is spoken by a computer voice or a picture of him is presented on a computer screen. These neurons thus seem to encode the semantic content of a stimulus, regardless of the sensory modality through which it is delivered. Previous work has predominantly focused on individual neurons that were selected based on their strong response to a particular stimulus using rather conservative statistical criteria. Those studies stressed that concept cells encode a single, concrete concept in an all-or-nothing fashion. Here, we analysed the neuronal code on the level of the entire population of neurons without any preselection. We conducted representational similarity analyses (RSAs) and pattern classification analyses of firing patterns evoked by visual stimuli (for example, a picture of an apple) that could be grouped into semantic categories on multiple levels of abstraction (‘fruit’, ‘food’, ‘natural things’). We found that neuronal activation patterns contain information on higher levels of categorical abstraction rather than just the level of individual exemplars. On the one hand, the neuronal code in the human MTL thus seems well suited to generalise semantic knowledge to new situations; on the other hand, it could also be responsible for the generation of false memories.

## Introduction

Cognitive faculties enabling flexible adaption of behaviour are at the heart of the human species’ evolutionary success. Cognition operates on abstract representations of knowledge derived from prior experience [1]. Abstraction can have two separate but related meanings [2]. First, formation of a concept in semantic memory requires abstraction in the sense of generalisation across episodes. For example, the concept ‘dog’, a furry animal that barks, is learned by extracting regularities among multiple encounters with various exemplars of dogs. Second, abstraction can also refer to the extraction of meaning from sensory input in a single instance of perception. Abstraction in the latter sense ranges from lower, more concrete levels (e.g., labelling a percept as ‘terrier’) to intermediate levels (‘dog’) and high, superordinate levels (‘animal’). Abstraction both as a cross-episode generalisation and as an extraction of supramodal semantic information from sensory input are in constant interplay and shape episodic and semantic memory representations [3,4].

Our knowledge about semantic representations in the human brain is for the most part restricted to the cortex. Putative functional roles of involved neocortical regions correspond to sensory and/or motor features of an encoded concept [1,5]. Here, abstract categories such as, for example, living and nonliving things differ with respect to which portions of the neocortex are recruited for their encoding. Due to such macroscopic, topographical organisation of semantic representations in the neocortex, these representations can be investigated with rather coarse imaging techniques such as functional magnetic resonance imaging [5]. Large strides have also been made in elucidating the neuronal code of object and face recognition along the ventral processing pathway of nonhuman primates leading up to highly abstract representation in monkey inferotemporal cortex and the amygdala [6,7]. Next to categorical codes, influential approaches also entail mapping semantic concepts onto a multidimensional, semantic space along dimensions such as living–nonliving or abstract–concrete [2,8,9].

Investigating object recognition and semantic representations at the final stages of the ventral processing pathway in the human medial temporal lobe (MTL), including the amygdala, has been notoriously difficult. Investigation of neuronal representations in the human MTL at the relevant level of detail seems impossible with noninvasive imaging techniques because—unlike the neocortex—most MTL areas lack semantic topographical organisation [10,11]. Studies conducted in the setting of invasive epilepsy monitoring using additional microelectrodes to record action potentials of single units have been instrumental for this purpose [10–15]. A seminal finding of these studies is that some MTL units responded in a selective and invariant manner to various images of a familiar person and even to their written and spoken name, suggesting that they encode the identity of that person and thus the contents of a concrete semantic concept in an all-or-none fashion [13,14]. However, further studies emphasised that MTL neurons can also respond to a wider range of stimuli in graded fashions in which sometimes more abstract semantic relations between stimuli can be identified such as, for example, membership to a broad category [9,14,15,16]. Thus, rather than all-or-nothing responses to specific concepts, it could be that neurons in the human MTL encode semantic features along continuous dimensions, resulting in ‘semantic tuning curves’. Or as Kornblith and Tsao [6] put it in the context of face-patches in primate IT, they are ‘[…] *measuring* faces, they are not yet explicitly *classifying* them’.

Previous human single unit studies often preselected units based on rather conservative response criteria, which may have resulted in a potential overestimation of all-or-none responses to individual semantic concepts. In the current study, in contrast, we analyse representations at the level of the entire population of units we record from. By doing so, we investigate how and at what level of abstraction semantic information conveyed by visual input is encoded in activity of single units in the human MTL. In contrast to previous studies, we consistently used the same set of images across sessions and patients, and the images could be grouped at multiple levels of abstraction. This procedure, in combination with a large sample of epileptic patients, allowed us to record neuronal responses for each image in a population of neurons unprecedented in size. Using this procedure, we could characterise and compare the nature of representations and their level of abstraction at a population level for different regions of the MTL.

## Results

Subjects (*N* = 25; 59 sessions) were bilaterally implanted with depth electrodes for seizure monitoring in the amygdala, hippocampus, entorhinal cortex, and parahippocampal cortex. Subjects were presented with visual stimuli depicting objects from 10 semantic categories consisting of 10 exemplars each (100 images, 10 trials each). The subjects’ task was to indicate by button press whether a man-made or natural object was depicted. As expected, this task was very easy as reflected by high accuracy (median = 97.62%, IQR = 2.25%) and short reaction times (median = 669 ms, IQR = 146 ms).

**Fig 1:**
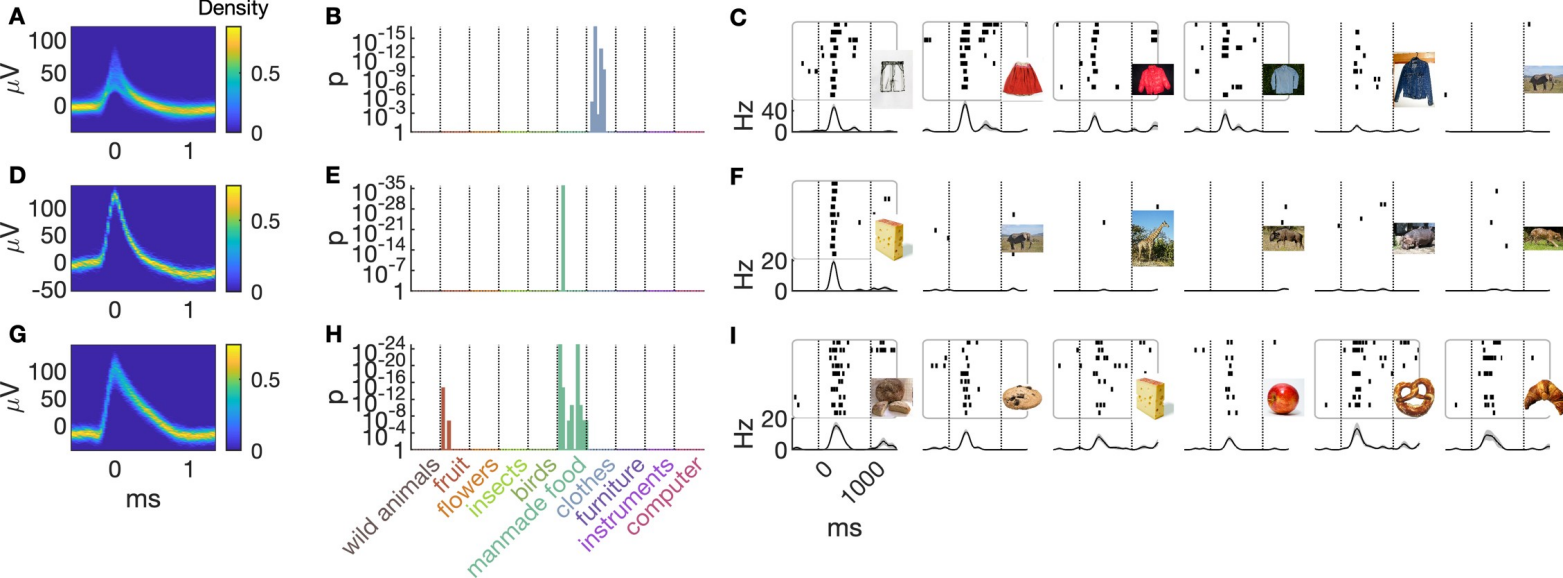
Single neurons respond selectively to a few stimuli in the set. Each row corresponds to data from one unit. The unit in the first row was recorded in the left anterior hippocampus. Units in rows two and three were recorded in the right amygdala. The three units were recorded in three different patients. (A, D, G) Spike shapes are depicted as temperature-scaled density plots. (B, E, H) Bar height in the histogram indicates the strength of neuronal responses to individual stimuli colour-coded according to superordinate semantic category. (C, F, I) Raster plots (stimulus onset: 0 s) depict the six most significant responses (grey rectangles indicate that the spiking pattern is considered as neuronal response to the stimulus; see Neuronal response test section in Materials and methods). Note that the images displayed here are different from the ones we actually used to prevent ostensible copyright infringement. Data and scripts underlying this figure as well as the image files we actually used are deposited here: https://github.com/rebrowski/abstractRepresentationsInMTL.

**Fig 2:**
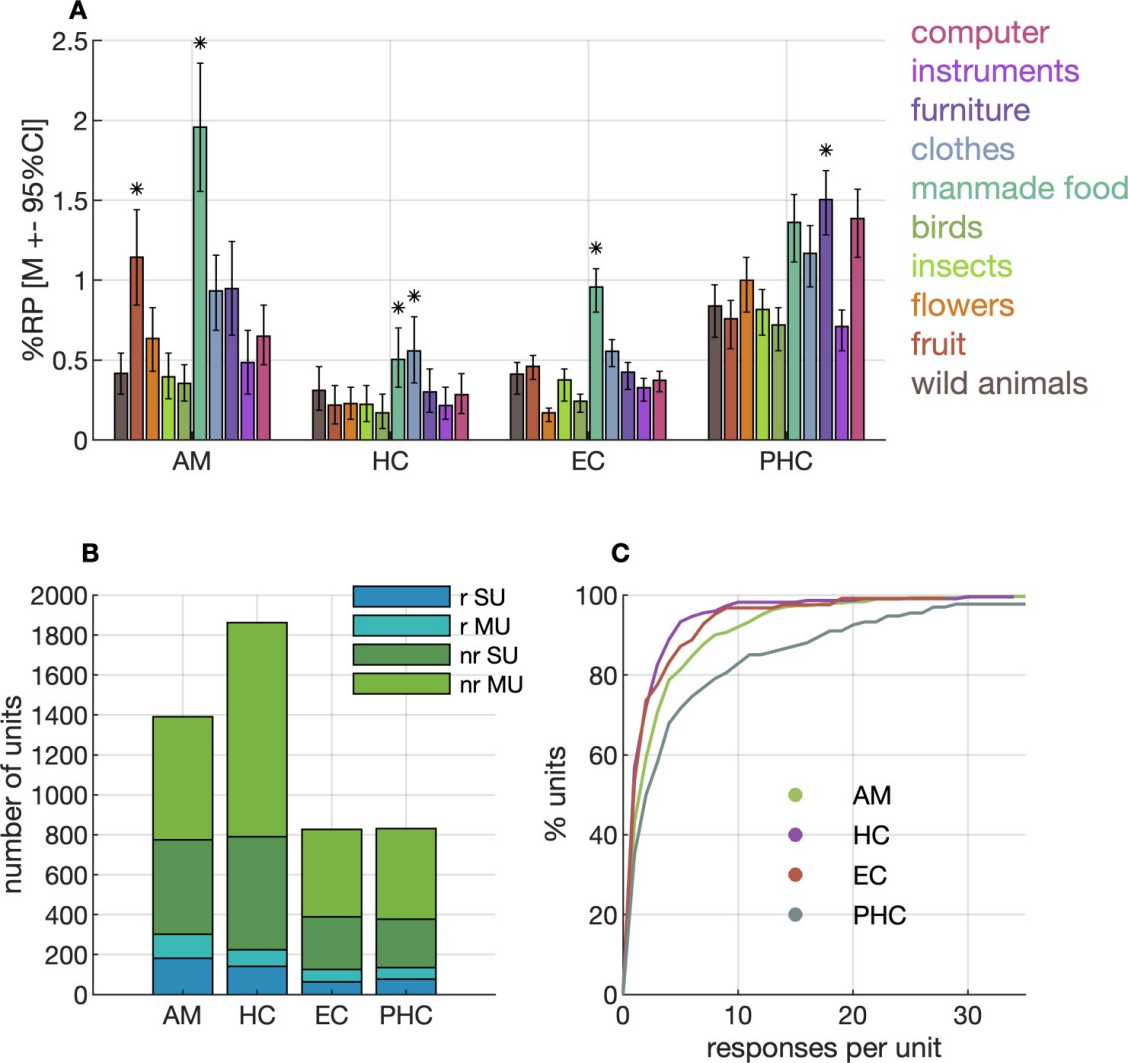
Response probabilities (RPs) and selectivity. (A) RPs (see Calculation of response probabilities section in Materials and methods) in percent across different categories and anatomical regions. Error bars indicate 95% CIs estimated by a subsampling procedure (see Calculation of response probabilities section in Materials and methods). Asterisks on top of a bar indicate a significant deviation of RP from RP of all other categories in a region (Fisher’s exact test, Bonferroni-corrected *α* = $\frac{0.05}{40}$). (B) Overview of SUs and MUs and their responsiveness to at least one of the stimuli. (C) Cumulative distributions of neuronal response selectivity, that is, the number of response-eliciting images identified per unit, truncated after 35 on the x-axis. Data and scripts underlying this figure are deposited here: https://github.com/rebrowski/abstractRepresentationsInMTL. AM, amygdala; CI, confidence interval; EC, entorhinal cortex; HC, hippocampus; MU, multiunit; nr, nonresponding; PHC, parahippocampal cortex; r, responding; RP, response probability; SU, single unit.

We first analysed our data by classifying units into responsive and nonresponsive, according to an established criterion (see Neuronal response test section in Materials and methods) as in previous studies [12,13] (Figs 1 and 2). Our analyses confirm that some units in the MTL respond to only a few stimuli in the set (Fig 1). We recorded from a total of 4,917 units, 2,009 of which were classified as single units (41%). In the amygdala, we found 1,392 units (656 single units [47%]), in the hippocampus 1,863 units (706 single units [38%]), in the entorhinal cortex 828 units (328 single units [40%]), and 831 units (319 single units [38%]) in the parahippocampal cortex (Fig 2B). A subset of 785 units responded with increased firing rates to at least one of the 100 stimuli (see Neuronal response test section in Materials in methods; Fig 2B). Selectivity as determined by the number of response-eliciting stimuli for a given neuron was similar in the entorhinal cortex, amygdala, and hippocampus but was markedly lower in the parahippocampal cortex [12] (Fig 2C). Some units responded very selectively, sometimes to only one of the stimuli in the set (Fig 1D–1F). In the amygdala, this was the case in 43% of the responsive units, in the hippocampus 57%, and in the entorhinal cortex 54%. This number was markedly lower in the parahippocampal cortex, namely, 35%. When units responded to multiple stimuli, the response-eliciting stimuli were often from the same semantic category (Fig 1A–1C and 1G–1I).

We also calculated the probabilities with which images from a given category elicited a neuronal response, separate for each anatomical region in the MTL. To this aim, we computed the number of significant responses across all units and divided this number by the total number of stimuli and the number of units. Observed response probabilities ranged between approximately 0.25% and 2% across anatomical regions and stimulus categories (Fig 1D). Neurons responded more frequently to food stimuli than to stimuli of other categories, which was especially prominent in the amygdala and, to a lesser degree, also in the hippocampus and entorhinal cortex (Fig 2A).

**Fig 3:**
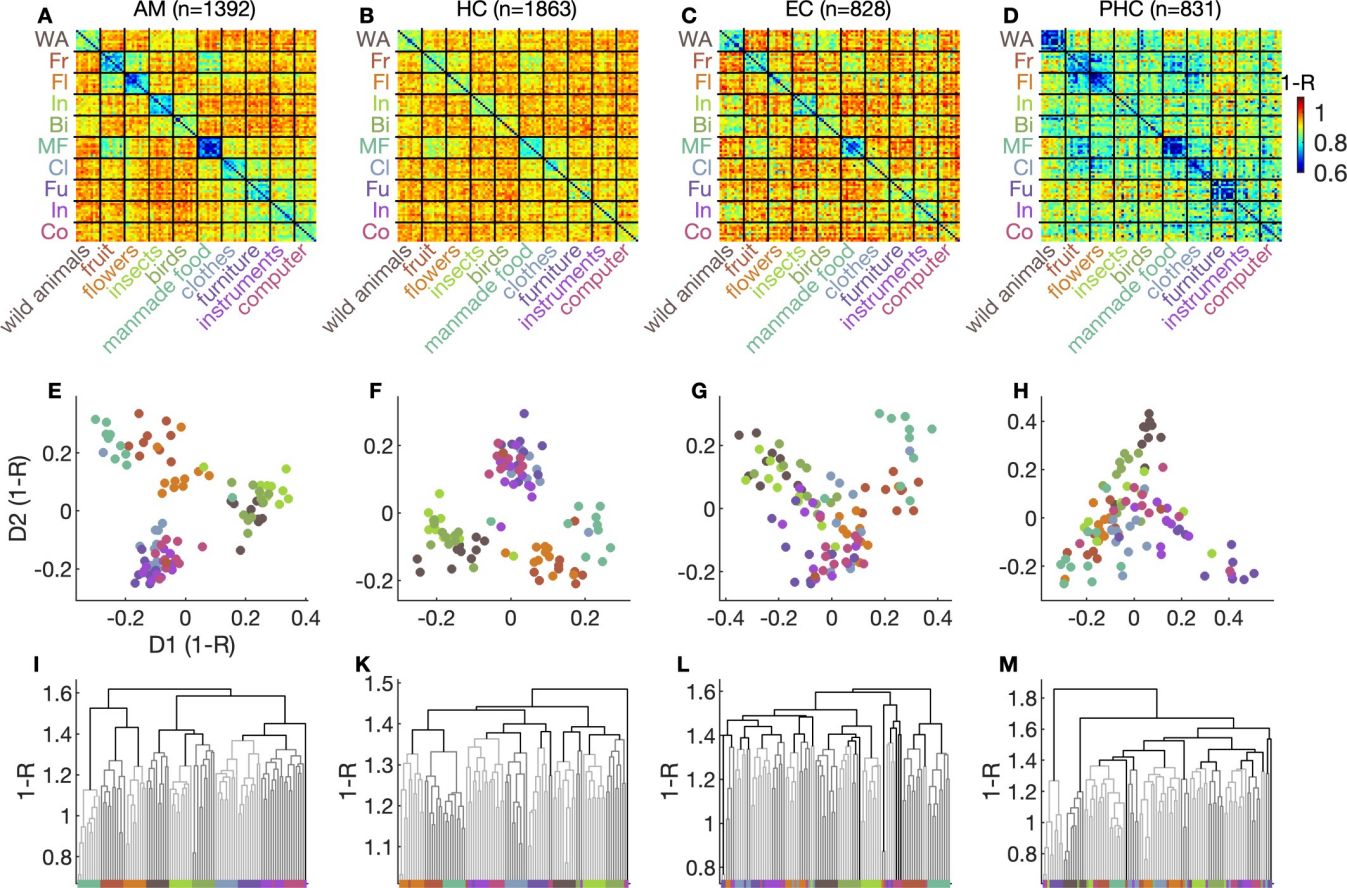
Abstract semantic features determine representational similarity at the population level. (A–D) Representational dissimilarity matrices showing the distance between two stimuli quantified as 1 –Pearson’s correlation coefficient *R* for the response activity of all recorded units. (E–H) Exemplars in two-dimensional space derived from multidimensional scaling of dissimilarity. (I–M) Dendrograms generated from automated hierarchical clustering. See S3 Fig for dendrograms spanning the full page width in each region. Data and scripts underlying this figure are deposited here: https://github.com/rebrowski/abstractRepresentationsInMTL. AM, amygdala; Bi, birds; Cl, clothes; Co, computer; D1, dimension 1; D2, dimension 2; EC, entorhinal cortex; Fl, flowers; Fr, fruit; Fu, furniture; HC, hippocampus; In (green), insects; In (purple), instruments; MF, manmade food; PHC, parahippocampal cortex; WA, wild animals.

Going beyond analyses of responsive versus nonresponding units, we next looked at responses of the whole population of units we recorded from. With these analyses, we find that population activity is determined by abstract, semantic features of the stimuli. We investigated population activity by representational similarity analyses (RSAs) [9,17,18]. To this aim, we quantified each neuronal response to a stimulus using a single *Z* score that expressed average firing across all trials of a stimulus in the 1,000 ms after stimulus onset, normalised using the distribution of baseline firing rates (−500 to 0 ms relative to stimulus onset) across all trials. The population response to a stimulus thus corresponded to a population vector of *Z* scores from all units in a given region. Representational dissimilarity (i.e., distance) between two stimuli was then quantified as 1 − Pearson’s correlation coefficient of their population vectors. Representational dissimilarities are displayed as matrices of colour-coded distance between all pairs of stimuli (Fig 3A–3D). Representational dissimilarity analyses showed that population firing patterns evoked by stimuli of the same category were more similar than those evoked by stimuli from different categories in all anatomical regions (Fig 3A–3D; all *p* < 10^−5^; random permutation test, Inference statistics on representational dissimilarity and confusion matrices section in Materials and methods).

To elucidate potential principles on higher levels of abstraction, we applied multidimensional scaling (Fig 3E–3H) and automated hierarchical clustering (Fig 3I–3M, S3 Fig) to these dissimilarity matrices. Remarkably, inspection of dendrograms obtained from hierarchical clustering revealed that the preconceived assignment of stimuli to superordinate categories was almost perfectly reflected in representational dissimilarity of the recorded population activity in the amygdala and hippocampus (Fig 3I and 3K). That preconceived categories matched information present in neuronal representations is evidenced by the sorting on the x-axis of the dendrograms. Perfect correspondence between neuronal similarity and category membership is indicated in that all exemplars of a category line up next to one another on the x-axis after sorting according to similarity. This is the case for all but two categories in the amygdala, in which only one exemplar of the ‘computer’ category ends up closer to other exemplars from the ‘musical instruments’ category. A similar pattern of exemplar sorting is evident in the hippocampus, whereas this was not the case in the entorhinal and parahippocampal cortex (Fig 3L and 3M). RSAs for units that did not show a response according to any of the stimuli in our set (according to the statistical response criterion used in this and previous studies) showed similar patterns of similarity (S1 Fig). Consequently, representational similarities of nonresponding units alone are statistically significantly higher for within- versus between-category pairs (all *p* < 10^−5^; see ‘Inference statistics on representational dissimilarity and confusion matrices’ section in Materials and methods), suggesting that even small variations in firing rate of MTL units contain considerable amounts of information at an abstract, categorical level.

Representations clustered beyond our preconceived categories in a highly abstract but meaningful way. Abstract semantic clusters of representational similarity emerging from neuronal representations are visualised by the dendrograms resulting from hierarchical clustering (Fig 3I–3K) and by projections of multidimensional scaling onto a two-dimensional space (Fig 3E–3H). In the amygdala, we saw a food cluster that consisted of all exemplars of man-made food and fruit categories. This food cluster becomes evident in that exemplars from the preconceived categories of ‘man-made food’ and ‘fruit’ are close together in the 2-dimensional projection generated by multidimensional scaling (Fig 3E). An animal cluster entailed exemplars of wild animals, birds, and insects. The categories of all man-made objects together constituted a further cluster. In the hippocampus, we additionally observed a clear separation between man-made and natural objects. This separation becomes evident when one draws a diagonal from top left to bottom right in Fig 3F that almost perfectly separates manmade from natural exemplars. Such clearly semantic principles governing representational similarity at a high level of abstraction were less evident in the entorhinal and parahippocampal cortex.

To assess whether low-level physical image similarity could have been responsible for these findings, we calculated four widely used statistics to compare physical properties of two images, namely, the Euclidean distance, the mean squared error, the peak signal-to-noise value, and the structural similarity index. We then performed analyses analog to the ones shown in Fig 3 using these image similarity measures (S2 Fig). These analyses showed no emergence of higher-order grouping of images according to abstract semantics as was the case for the neural data (Fig 3). Therefore, we conclude that low-level physical similarity cannot account for the findings of representation similarity in our neuronal response patterns.

**Fig 4:**
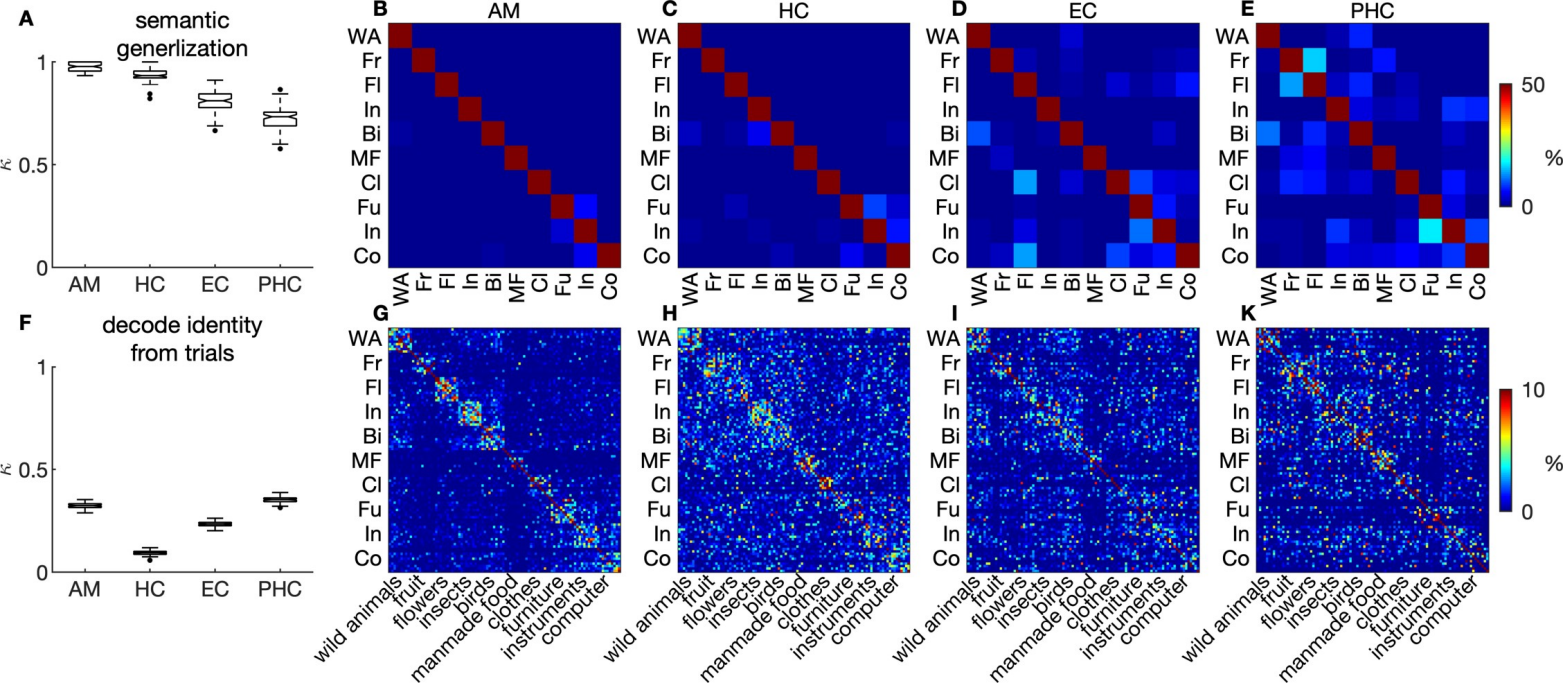
Pattern classifier algorithms learn abstract semantic information. (A–E) Classifiers were trained to classify the superordinate category from *Z* scored responses to half of the stimuli per category and tested out of sample on the other half. Classification performance on 100 random divisions of data into training and test set is indicated in box plots (Cohen’s κ). (B–I) Confusion matrices (rows: correct label; columns: predicted label). (F–K) Classifiers were trained on half of the trials per stimulus to predict individual stimulus identity and tested out of sample on the other half of trials. Colour codes extend to maximally 50% (B–E) and 10% (G–K) for display purposes. Values higher than these maxima (for example, squares on the main diagonal) are not resolved in favour of making the patterns in off-diagonal areas more clearly visible. Data and scripts underlying this figure are deposited here: https://github.com/rebrowski/abstractRepresentationsInMTL. AM, amygdala; Bi, birds; Cl, clothes; Co, computer; EC, entorhinal cortex; Fl, flowers; Fr, fruit; Fu, furniture; HC, hippocampus; In, insects; In, instruments; MF, manmade food; PHC, parahippocampal cortex; WA, wild animals.

Abstraction comes at a trade-off between generalisation of knowledge to new situations and confusion between similar exemplars. We used the population responses described above to train pattern classifiers (multiclass support vector machine models; see Decoding of stimulus identity and category section in Methods and materials). A classifier was trained on the population responses of half the stimuli per category to predict the category label and was then tested out of sample on population responses of the other half of stimuli. This procedure was repeated 100 times with random divisions of the data into training and test sets. Successful generalisation to untrained stimuli was indicated by highly accurate out-of-sample classification of category labels from population responses (Fig 4A; for separate analyses for each subject, collapsing across anatomical regions, see S4 Fig). Generalisation was best using population responses from amygdala units, intermediate using hippocampal and entorhinal units, and lowest using parahippocampal units. Nevertheless, generalisation exceeded chance performance in all MTL regions by far.

To assess performance in classifying individual stimuli, we calculated *Z* scored population responses of unit firing for each trial in the same manner as described above. Pattern classification algorithms were then trained on population responses of half of the trials for each stimulus and tested out of sample on the other half. Again, out-of-sample performance was assessed in 100 random divisions of the data into training and test set. Classification performance exceeded chance level in all regions of the MTL (Fig 4F). Interestingly, we found a systematic pattern of misclassifications when inspecting confusion matrices (Fig 4G–4K). Confusion matrices cross-tabulate the number of classifier outcomes by predicted stimulus label in columns and true stimulus labels in rows. These analyses show that pattern classification algorithms trained to decode individual stimulus identity more often confused stimuli from the same versus different superordinate categories (Fig 4F–4K; all regions *p* < 10^−5^, permutation test; see ‘Inference statistics on representational dissimilarity and confusion matrices’ section in Materials and methods; for analogous analyses separately for each subject but collapsing across anatomical regions, see S5 Fig).

## Discussion

Taken together, our results provide a novel perspective on how information is encoded in the human MTL. We demonstrate that despite selective tuning of individual neurons to only a few stimuli in the set, activity at the population level is determined by information with a high degree of semantic abstraction. We find that population activity is similar in response to exemplars of the same category and that response pattern similarity extends to highly abstract semantic categories. Pattern classification results show high levels of semantic abstraction, which, on one hand, can be useful for successful generalisation of knowledge to novel situations. On the other hand, semantic abstraction comes at the cost of confusion between semantically similar stimuli.

With respect to neuronal representations in the MTL, we demonstrate a semantic code that spans multiple layers of abstraction emerging at the population level. This perspective may aid to reconcile disparate findings from previous studies investigating response properties of individual units [11,13,16]. Some have concluded that unit activity encodes concrete concepts such as, for example, a person’s identity [13,14]. Others postulate superordinate category membership as a decisive feature driving unit activity [16,19]. Our study may reconcile these views as population-level analyses show that encoded information spans across multiple levels of abstraction ranging from the concrete exemplar level to the level of preconceived semantic categories and beyond. Pattern classification analyses demonstrate that information on the exemplar and superordinate categorical level can both be decoded from population activity, whereas categorical information seems predominant. These aspects may not become apparent when looking at response profiles of individual units and underscore the importance of analyses at the population level.

Furthermore, our data refine the view on sparseness of coding in the human MTL. Hallmark human single unit studies suggest that very few concepts drive activity in one single neuron [13,14,20]. In fact, considerably more than 50% of responsive units were found previously to respond to only one out of approximately 100 stimuli [12]. This is true in the amygdala, hippocampus, and entorhinal cortex, whereas selectivity is lower in the parahippocampal cortex [12]. These findings led to the conclusion that the MTL uses a very sparse, almost ‘grandmother cell’-like code [21]. Although some units in our data set indeed only fired in response to one stimulus in the set, the overall selectivity in our study was lower (see Fig 1F) than reported earlier [12,20]. Previous studies used stimulus sets that were tailored to the patients’ interests, depicting relatives, preferred celebrities, and job- and hobby-related objects [12,13]. The aim in these studies was to screen for response-eliciting stimuli using a wide range of different concepts, likely resulting in rather low semantic feature overlap between stimuli. Our current stimulus material had a systematic semantic structure because images were grouped into categories of semantically related exemplars. Assuming that unit activity is determined by a rather narrow ‘semantic tuning curve’, we would indeed expect that neurons fire less selectively when ‘semantic distance’ between stimuli is sufficiently low. Thus, semantic relatedness between stimuli in a set seems likely to influence estimates of sparseness of unit responses in the MTL.

Two previous studies have applied RSAs to single units in the human MTL. First, in 2011, Mormann and colleagues [17] used RSA in combination with images that could be grouped into 3 categories, namely, persons, animals, and landmarks. This study found that the amygdala is preferentially activated by animal stimuli but did not investigate the semantic nature and level of abstraction in amygdala unit activity. Furthermore, a 2015 paper again by Mormann and colleagues [18] used RSA to show that units in the amygdala encode face identity rather than gaze direction. Again, analyses focused on the amygdala, and semantic abstraction could not be assessed because stimuli consisted of pictures of faces with gazes pointed in different directions.

Furthermore, the notion of an all-or-nothing response behaviour as implied in earlier studies (for example, [13,20]) should be critically reevaluated. Obviously, response behaviour strongly depends on the exact definition of the statistical response criterion employed. Previous studies have used a rather conservative response criterion and tended to regard any activity not meeting this criterion as background noise [12,13,20]. Our analyses demonstrate that even after excluding all neurons that showed statistical responses to any of the presented stimuli, semantic category information is still present in the population activity of the ‘nonresponsive’ neurons. Thus, such subthreshold responses according to this criterion are likely to carry relevant information about the presented stimulus. For example, looking at Fig 1 A and 1C, we see such subthreshold responses. Here, the units clearly prefer stimuli from one category (for example, clothing items in case of 1A). Within this category, however, some images drive spiking activity more strongly than others. The jean jacket in Fig 1A is the fifth-most response-eliciting stimulus for that unit but falls short of being classified as a response by the criterion we use, as indicated by the absence of a grey box around the respective raster plot. In view of the other response-eliciting stimuli, we would probably conclude that this might be a true but subthreshold response. Arguably, there are some units in the data set for which we find only such subthreshold responses because the near-optimal stimuli for these units were not in our set. It thus seems that these subthreshold units carry a significant amount of categorical information at the population level. Together, these results suggest that neurons do not encode the identity of a concept in an all-or-none fashion but rather that firing patterns may be best described as graded with the assumption of an underlying ‘semantic tuning curve’.

The high levels of abstraction in population activity observed in this study could also suggest a single-unit mechanism in the MTL for the generation of false memories. Classically, false memories are studied by presenting semantically related words for study, for example, ‘giraffe’, ‘lion’, ‘elephant’, or ‘tiger’, followed by a recognition memory test requiring old–new judgments of old words (for example, ‘lion’), as well as new words that were either semantically related (‘leopard’) or unrelated (‘keyboard’) to the studied words [22]. False memories manifest in more frequent old judgments to new words with high versus low semantic relatedness [22,23]. Overlap of recruited neocortical regions corresponds to semantic feature overlap between studied and new words, which, in turn, is correlated with false-memory likelihood [24]. However, it seems likely that overlap in recruitment of neocortical regions is in fact the consequence of ‘false’ reinstatement initiated by the hippocampus rather than the cause of false memories [24,25]. The hippocampus has been shown to be equally active during false and true memories in humans [26], and optogenetic activation of neurons in the rodent hippocampus has been shown to trigger reinstatement of ‘false’ contextual fear memories [25]. Our data suggest that confusion between semantically similar stimuli is facilitated by the abstract semantic code utilised by neurons in the hippocampus, and thereby provides a link between human behavioural and functional magnetic resonance imaging versus rodent optogenetic studies of false-memory generation [22,24–26].

The combination of RSA and pattern classification applied to our single neuron data reveals novel insights about the neuronal code for semantics in the MTL. Although we think that the decoding of semantic generalisation (top row of Fig 4) and the RSA analyses (Fig 3) convey similar aspects of the data, the decoding results are by no means a trivial consequence of the RSA analyses. First, the decoding analyses allow for a comparison of decoding accuracy for exemplar versus category decision. Second, the fact that confusions within category are more frequent than those across category offers a mechanistic explanation for the generation of false memories. Both of these points do not become apparent from the RSA results alone. These RSA results, in turn, show higher-order organising principles of semantic information in populations of single neurons in the MTL.

Our study also contributes to the understanding of neuronal representations in the amygdala. We found a preference of amygdala units for stimuli depicting food items, which dovetails with findings of a potential role of the amygdala in modulating food consumption recently reported in rodents [27] and with views of the role of the amygdala in processing positive and negative value as well as relevance of stimuli [28,29]. However, human amygdala units have also been shown to preferably respond to animals [17], to be involved in processing of faces and parts of faces [30,31], and to encode the intensity of emotion in facial expressions [32]. More generally, the amygdala has been hypothesised to be involved in social cognition [31]. It is noteworthy that we do not see a preference for stimuli depicting animals in the amygdala as reported by Mormann and colleagues (2011) [21]. Response probabilities of animal stimuli in our study are comparable to this study (approximately 1%). Mormann and colleagues (2011), however, compared animal stimuli to pictures of persons, landmarks, and objects, which all had significantly lower response probabilities (approximately 0.2%). Thus, we may not see a preference for animals because the categories to which we compare them (for example, food, plants, musical instruments, etc.) are different. It may help to reconcile this broad range of findings to consider that the amygdala is a complex and heterogeneous structure consisting of multiple nuclei involved in a wide range of different functions [33] and that the exact location of microwires with respect to these nuclei cannot be determined with sufficient accuracy in human subjects.

Finally, our data connect to notions of hierarchical processing within the MTL. Strong tuning to highly abstract semantics has been found in the hippocampus and the amygdala. Both regions receive highly processed, supramodal input [12,33,34]. The use of a highly abstract semantic code appears plausible to aid in attributing value and relevance of stimuli, a function hypothesised to occur in the amygdala [28]. In the hippocampus, high levels of abstraction may facilitate efficient and sparse representations of large amounts of information encoded in neocortical firing patterns for subsequent encoding of episodic memories [35–37]. In contrast, abstract semantic representations were less pronounced in parahippocampal and entorhinal neurons. This finding connects with views that these structures are situated at a lower stage of the processing hierarchy within the MTL [12,34,38]. Here, the parahippocampal cortex acts as an input region for higher MTL regions. Parahippocampal neurons fire earlier, less selectively than in other MTL regions [12], and display a preference for images with spatial layout of visual input [10]. Similarly, the entorhinal cortex relays reciprocal connections between hippocampus and neocortex [34] and has also been found to be involved in spatial processing in humans [39,40].

## Materials and methods

### Participants

A total of 25 epileptic patients (9 female) aged 19 to 62 y (M = 38, SD = 13) were implanted with depth electrodes for chronic seizure monitoring. Their average stay on the monitoring ward was 7 to 10 d.

### Ethics statement

The study was approved by the Medical Institutional Review Board of the University of Bonn (accession number 095/10 for single-unit recordings in humans in general and 245/11 for the current paradigm in particular) and adhered to the guidelines of the Declaration of Helsinki. Each patient gave informed written consent.

### Task and stimuli

One hundred images from 5 man-made and 5 natural categories of 10 exemplars each were selected as stimuli. The experiment was subdivided into 10 runs. One run entailed sequential presentation of all 100 images in the set in pseudorandom order. A trial entailed the presentation of a blank screen for a variable duration (200–400 ms) and a fixation dot for 300 ms, followed by the image that stayed on screen until the subject responded with a button press. Subjects were instructed to press the left or right arrow key if the image on the screen depicted a man-made or natural object, respectively.

### Electrophysiological recordings and spike sorting

Nine microwires (8 high-impedance recording electrodes, 1 low-impedance reference; AdTech, Racine, WI) protruding from the shaft of the depth electrodes were used to record signals from MTL neurons. Signals were amplified and recorded using a Neuralynx ATLAS system (Bozeman, MT). The sampling rate was 32 kHz, and signals were referenced against one of the low-impedance reference electrodes. Spike sorting was performed using wave_clus [41] in 33 sessions and using Combinato (https://github.com/jniediek/combinato) [42] in 26 sessions. Different spike-sorting routines were used as the reported paradigm also served as a procedure to screen for response-eliciting stimuli in the morning of a day of testing. Therefore, manual optimisation of spike sorting was performed immediately after recording. The lab as a whole switched to using Combinato for reasons unrelated to the reported research.

A total of 5,033 units resulted from spike sorting, 4,917 of which were recorded in one of the anatomical regions considered (amygdala, hippocampus, entorhinal cortex, and parahippocampal cortex). The number of microwires per patient was on average 71.60 (SD = 21.32) and ranged from 32 to 96. On average, we recorded 1.38 units per microwire (SD = 0.44). These values ranged from 0.41 to 2.24 across all 59 sessions.

### Neuronal response test

To determine whether a unit responded with increased spiking activity to one of the stimuli in the set, we calculated a binwise rank-sum test described earlier [12]. We obtained spike counts in 19 overlapping 100 ms bins ([0:100:1,000] and [50:100:950] ms after stimulus onset) for each trial in which a given image was presented. We computed 19 rank-sum tests, each of which compared the distribution of spike counts of one of the 19 bins against the distribution of spike counts in a baseline interval (−500 to 0 ms) of all trials in a session. The resulting 19 *p*-values were corrected for multiple comparisons using the Simes procedure. A stimulus was classified as eliciting a neuronal response in a unit when one or more of these 19 *p*-values was lower than *α* = 0.001. Furthermore, we considered only increases in firing rates. Also, neuronal responses were only considered as such if at least one spike in the response period was recorded in more than 5 out of the 10 trials per image and if the average firing rate during the response window (0 to 1,000 ms) was above 2 Hz.

### Calculation of response probabilities

We counted the neuronal responses across all sessions, separate for superordinate category and anatomical location. To make these values comparable across anatomical regions and with previous work [17], we calculated response probabilities by normalising these counts to the number of units in an anatomical region and the total number of stimuli presented (100). Response probabilities were calculated for each of the four anatomical regions of interest. They thus represent the empirical probability that a unit in a given anatomical region will respond to a stimulus from a given semantic category.

We obtained measures of dispersion of these response probabilities by using a subsampling procedure. We drew 2,000 random subsamples of 700 units without replacement from each region and derived 95% confidence intervals from the resulting distributions of response probabilities for each category of stimuli.

A Fisher’s exact test on the response probabilities was conducted for each category and each anatomical region. To this aim, data were arranged in a 2 × 2 contingency table of the frequencies of significant and nonsignificant neuronal responses in a superordinate category of interest, and the frequency of significant and nonsignificant neuronal responses in all other superordinate categories.

### Representational dissimilarity analyses

To assess the dissimilarity between neuronal representations of stimulus categories, firing rates during the response period (0 to 1,000 ms after stimulus onset) of each stimulus were expressed as *Z* scores using the mean and standard deviation of firing rates in a base line interval ranging from −500 ms to stimulus onset (0) across all trials. These *Z* scores were arranged in a matrix of *N*_*S*_ × *N*_*U*_, where *N*_*U*_ is the number of units recorded and N_S_ the number of stimuli in the set (100). Representational dissimilarity between a pair of stimuli was calculated using 1 –Pearson’s correlation coefficient (1 − *R*) of the vectors of *Z* scores corresponding to the population activity evoked by the two stimuli in a pair [9,17]. To assess representational dissimilarity on the level of individual trials, we computed *Z* scores for each trial in the experiment. These *Z* scores were arranged in a matrix of *N*_*T*_ × *N*_*U*_, where *N*_*U*_ is the number of units recorded and *N*_*T*_ the number of trials during the paradigm (1,000).

Hierarchical clustering for dendrograms in Fig 3 was performed using unweighted average distance method on correlation distances.

### Decoding of stimulus identity and category

We used the matrices of *Z* scores described above (*N*_*T*_ × *N*_*U*_) to assess pattern classification performance. We used the function fitcecoc.m from MATLAB’s (MathWorks; www.mathworks.com) statistics and machine-learning toolbox. This function was used to train a multiclass, error-correcting output codes model of linear support vector machines for binary choices. Binary support vector machines were specified according to a ‘one versus all’ coding scheme in which for each binary classifier, one class is positive and the rest are negative. The classifier was trained to predict the label of stimulus identity from individual trials (*N*_*T*_ × *N*_*U*_). Out-of-sample performance was assessed for 100 pseudorandom divisions of the data into training and test set (50% holdout for test). To test for semantic generalisation to ‘unseen’ members of category, further classifiers were trained on the mean responses (*N*_*S*_ × *N*_*U*_) of half of the stimuli to learn category labels and tested on the other half of stimuli. Again, out-of-sample performance was assessed for 100 pseudorandom divisions of the data into training and test set. Classification performance was quantified by $Cohen\prime s\;\kappa =\frac{P_{O}-P_{C}}{1-P_{C}}$, where P_O_ is the observed agreement and P_C_ is chance agreement. S4 Fig and S5 Fig show these same analyses repeated separately for each subject but collapsing across regions.

### Inference statistics on representational dissimilarity and confusion matrices

To assess whether dissimilarity (1 − R) was significantly different within versus across exemplars of superordinate categories, we implemented a label-shuffling procedure. To this aim, we arranged dissimilarity between all pairs of stimuli in matrices of the format *N*_*S*_ × *N*_*U*_. Next, we selected a set of indices to the elements in these matrices that correspond to within-category dissimilarity. Another set of indices was selected corresponding to between-category dissimilarity. We then computed a Mann-Whitney U test with the hypothesis that within-category dissimilarity is lower than between-category dissimilarity. From this test we obtained a test statistic (rank-sum) of the original assignments of the labels (within- versus between-category dissimilarity) to the data. We repeated this test 10^5^ times with randomly shuffled assignments of labels to the data, that is, indices to the matrix corresponding to within- versus between-category pairs were randomised and hence mostly false. Of these 10^5^ tests with random labels, we saved the distribution of resulting test statistics (rank-sums). The reported *p*-values reflect the percentile of the test statistic that got the correct assignments of labels to the data within the distribution of test statistics derived with randomly relabelled data. The same procedure was carried out for the confusion matrices derived from pattern classification. Note that dissimilarity matrices were symmetric, whereas confusion matrices were not. We therefore computed statistics for dissimilarity on the triangular matrices only.

### Analyses and stimulus-delivery software

We used MATLAB and its statistics and machine-learning toolbox in combination with custom code for analyses of the data. Spike sorting of 33 sessions was done using wave_clus (https://github.com/csn-le/wave_clus) [41]. The remaining 26 sessions were sorted using Combinato [42] requiring Python (www.python.org). We used the psychtoolbox3 (www.psythoolbox.org) and octave (www.gnu.org/octave) running on a Debian 8 operating system (www.debian.org) on a standard laptop computer for stimulus delivery. All relevant data and custom code are available on https://github.com/rebrowski/abstractRepresentationsInMTL.git.

## Supporting information

S1 Fig Representational similarity of only nonresponding units shows similar patterns as for all units (cf. Fig 2). (A–D) Representational dissimilarity matrices showing the distance between two stimuli quantified as 1 − Pearson’s correlation coefficient (*R*) for the response activity of all recorded units. (E–H) Exemplars in two-dimensional space derived from multidimensional scaling of dissimilarity. (I–M) Dendrograms generated from automated hierarchical clustering. Data and scripts underlying this figure are deposited here: https://github.com/rebrowski/abstractRepresentationsInMTL.
(TIF)

S2 Fig Measures of picture similarity were computed for each pair of images and subjected to the same analyses as neuronal similarity (Fig 3). Picture similarities were calculated using the Euclidean distance (A, E, I), the mean squared error (B, F, K), the structural similarity index (ssi) (note that we display the ssi subtracted from the maximal ssi to achieve a measure of distance), and the peak signal-to-noise ratio (psnr) (again, we display max(pnsr) − pnsr to obtain distance rather than similarity). Data and scripts underlying this figure are deposited here: https://github.com/rebrowski/abstractRepresentationsInMTL. pnsr, peak signal-to-noise ratio; ssi, structural similarity index.
(TIF)

S3 Fig Dendrograms resulting from automated hierarchical clustering of representational dissimilarity (same as Fig 2I–2M). Data and scripts underlying this figure are deposited here: https://github.com/rebrowski/abstractRepresentationsInMTL. AM, amygdala; EC, entorhinal cortex; HC, hippocampus; PHC, parahippocampal cortex.
(TIF)

S4 Fig Semantic generalisation decoding, separately for each subject. Analog to Fig 4. Depicted are confusion matrices of decoding analyses based on data of each individual subject, collapsed across anatomical regions and sessions (see Decoding of stimulus identity and category section in Materials and methods). Decoders were trained to predict the category label of stimuli, trained on data of half of the stimuli in each category. Out-of-sample accuracies in 100 random subdivisions of data into training and test sets for each of the 25 subjects are depicted in the box plots beneath the confusion matrices. Note that boxes of decoding accuracies are above chance level (dotted line, 10%) in all subjects. Successful out-of-sample decoding on new exemplars of the category indicates an abstract semantic code implemented in the neuronal firing of MTL regions. Data and scripts underlying this figure are deposited here: https://github.com/rebrowski/abstractRepresentationsInMTL. MTL, medial temporal lobe.
(TIF)

S5 Fig Decoding of stimulus identity from trials per subject. Analog to Fig 4. Depicted are confusion matrices of decoding analyses based on data of each individual subject, collapsed across anatomical regions and sessions (see Decoding of stimulus identity and category section in Materials and methods). Decoders were trained to predict the label of stimuli and trained on data of half of the trials per stimulus. Out-of-sample accuracies in 100 random subdivisions of data into training and test sets for each subject are depicted in the box plots beneath the confusion matrices. Note that boxes of decoding accuracies are above chance (dotted line, 1%) in all subjects and that confusions between stimuli occur more often within rather than across category. Data and scripts underlying this figure are deposited here: https://github.com/rebrowski/abstractRepresentationsInMTL.
(TIF)

## Abbreviations

MTL: medial temporal lobe
RSA: representational similarity analysis
