# Correction: Representation of abstract semantic knowledge in populations of human single neurons in the medial temporal lobe

**DOI:** 10.1371/journal.pbio.3000753

**Published:** 2020-05-19

**Authors:** Thomas P. Reber, Marcel Bausch, Sina Mackay, Jan Boström, Christian E. Elger, Florian Mormann

The response window used to calculate Z-scored firing rates was incorrectly indicated as ranging from 0 ms to 1000 ms after stimulus onset. The correct range is 100 ms to 1000 ms post-stimulus. The response window definition for one analysis, namely the decoding analysis on individual trials, however, was performed using the incorrect definition of the response window. This analysis was re-run using the same response window definition as in the analysis with the averaged firing rates across trials of a stimulus, which resulted in minimal changes to Fig 4F–4K. Statistical analyses and the conclusions drawn from these analyses remain unchanged. The new data frames have been uploaded to the public repository with the data accompanying the paper (https://github.com/rebrowski/abstractRepresentationsInMTL)

**Fig 4:**
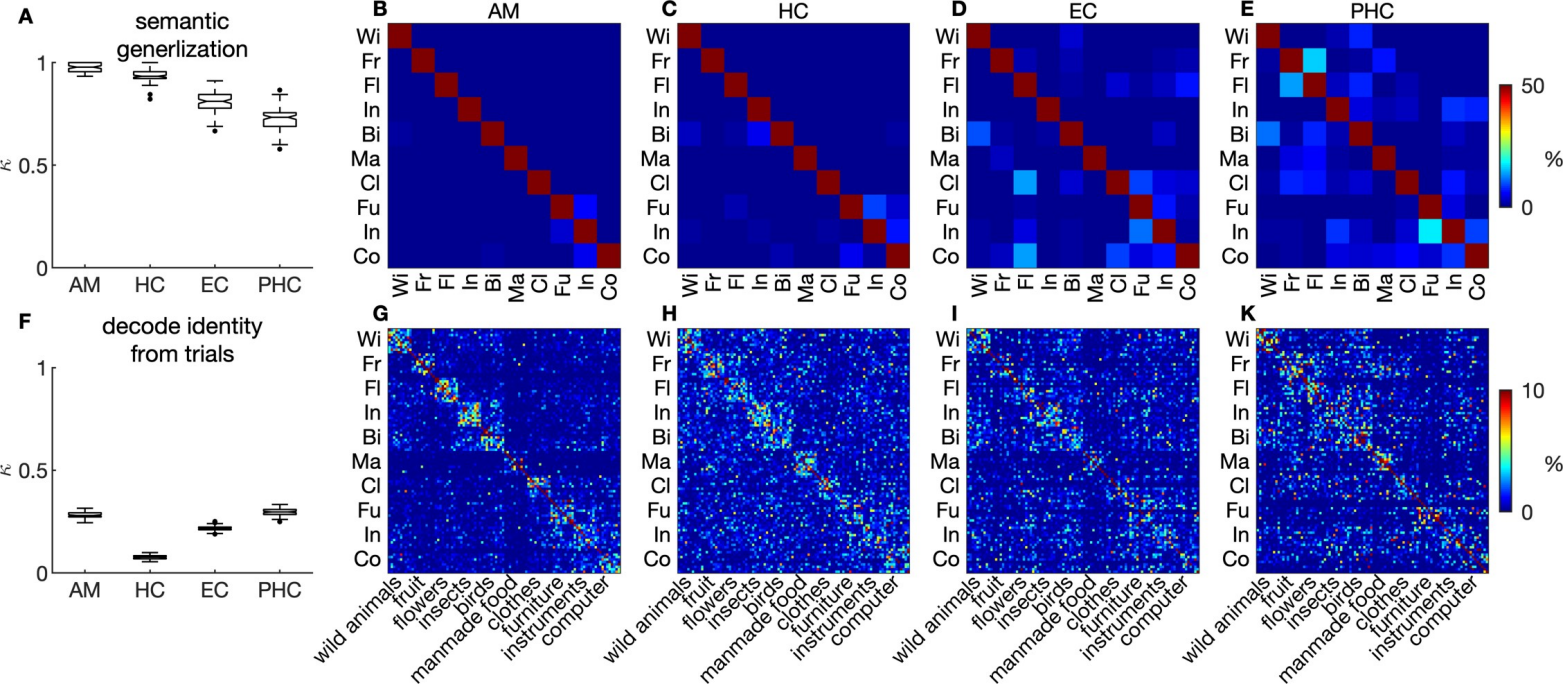
Pattern classifier algorithms learn abstract semantic information. (A–E) Classifiers were trained to classify the superordinate category from Z scored responses to half of the stimuli per category and tested out of sample on the other half. Classification performance on 100 random divisions of data into training and test set is indicated in box plots (Cohen’s κ). (B–I) Confusion matrices (rows: correct label; columns: predicted label). (F–K) Classifiers were trained on half of the trials per stimulus to predict individual stimulus identity and tested out of sample on the other half of trials. Colour codes extend to maximally 50% (B–E) and 10% (G–K) for display purposes. Values higher than these maxima (for example, squares on the main diagonal) are not resolved in favour of making the patterns in off-diagonal areas more clearly visible. Data and scripts underlying this figure are deposited here: https://github.com/rebrowski/abstractRepresentationsInMTL. AM, amygdala; Bi, birds; Cl, clothes; Co, computer; EC, entorhinal cortex; Fl, flowers; Fr, fruit; Fu, furniture; HC, hippocampus; In, insects; In, instruments; MF, manmade food; PHC, parahippocampal cortex; WA, wild animals.
