# Supplementary figures and images for: Representation of abstract semantic knowledge in populations of human single neurons in the medial temporal lobe
Source: PLoS Biol. 2019 Jun 3;17(6):e3000290. doi: 10.1371/journal.pbio.3000290 (PMC6564037; doi:10.1371/journal.pbio.3000290)

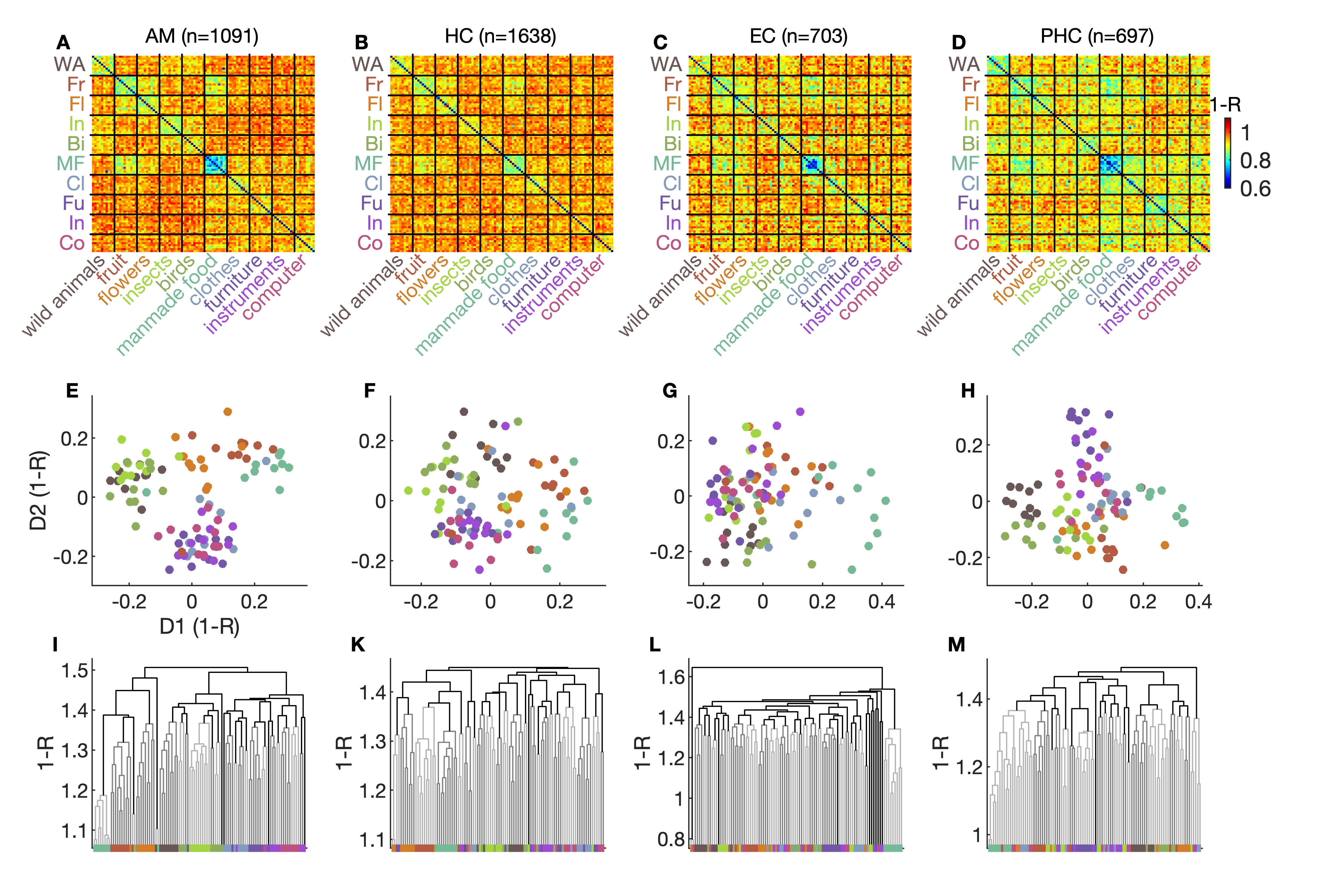

Supplement: S1 Fig — (A–D) Representational dissimilarity matrices showing the distance between two stimuli quantified as 1 − Pearson’s correlation coefficient (R) for the response activity of all recorded units. (E–H) Exemplars in two-dimensional space derived from multidimensional scaling of dissimilarity. (I–M) Dendrograms generated from automated hierarchical clustering. Data and scripts underlying this figure are deposited here: https://github.com/rebrowski/abstractRepresentationsInMTL. (TIF) [file pbio.3000290.s001.tif]

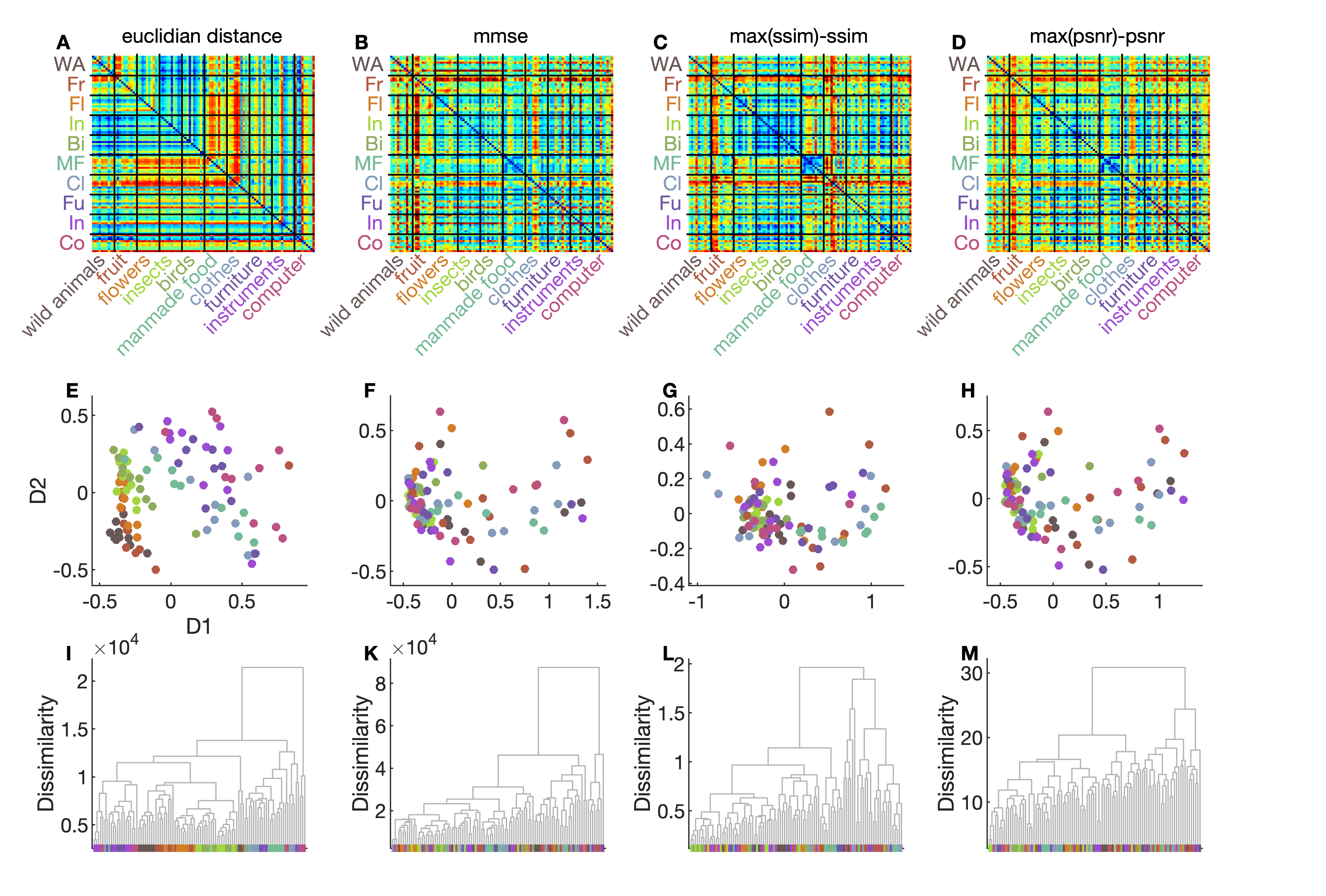

Supplement: S2 Fig — Picture similarities were calculated using the Euclidean distance (A, E, I), the mean squared error (B, F, K), the structural similarity index (ssi) (note that we display the ssi subtracted from the maximal ssi to achieve a measure of distance), and the peak signal-to-noise ratio (psnr) (again, we display max(pnsr) − pnsr to obtain distance rather than similarity). Data and scripts underlying this figure are deposited here: https://github.com/rebrowski/abstractRepresentationsInMTL. pnsr, peak signal-to-noise ratio; ssi, structural similarity index. (TIF) [file pbio.3000290.s002.tif]

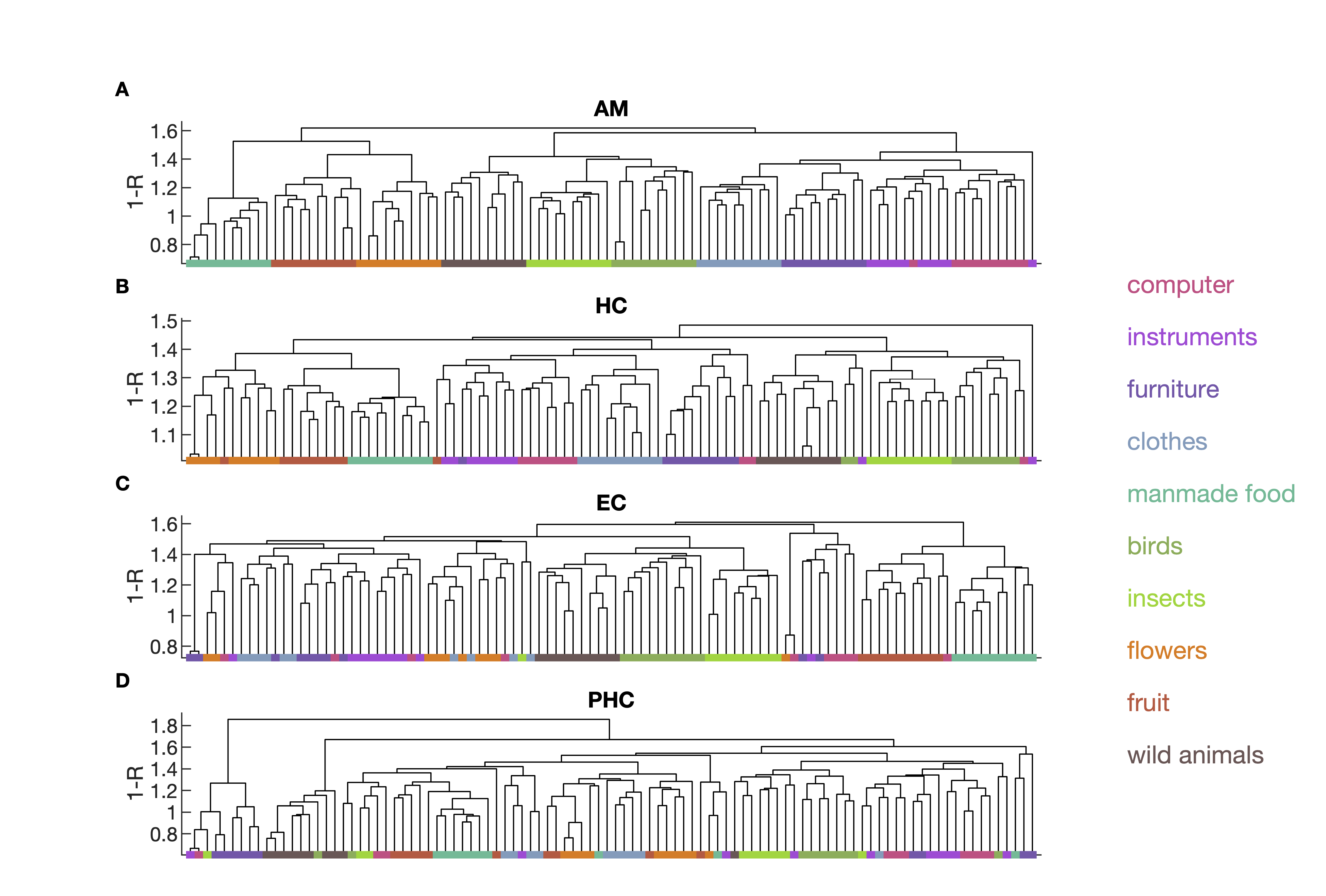

Supplement: S3 Fig — Data and scripts underlying this figure are deposited here: https://github.com/rebrowski/abstractRepresentationsInMTL. AM, amygdala; EC, entorhinal cortex; HC, hippocampus; PHC, parahippocampal cortex. (TIF) [file pbio.3000290.s003.tif]

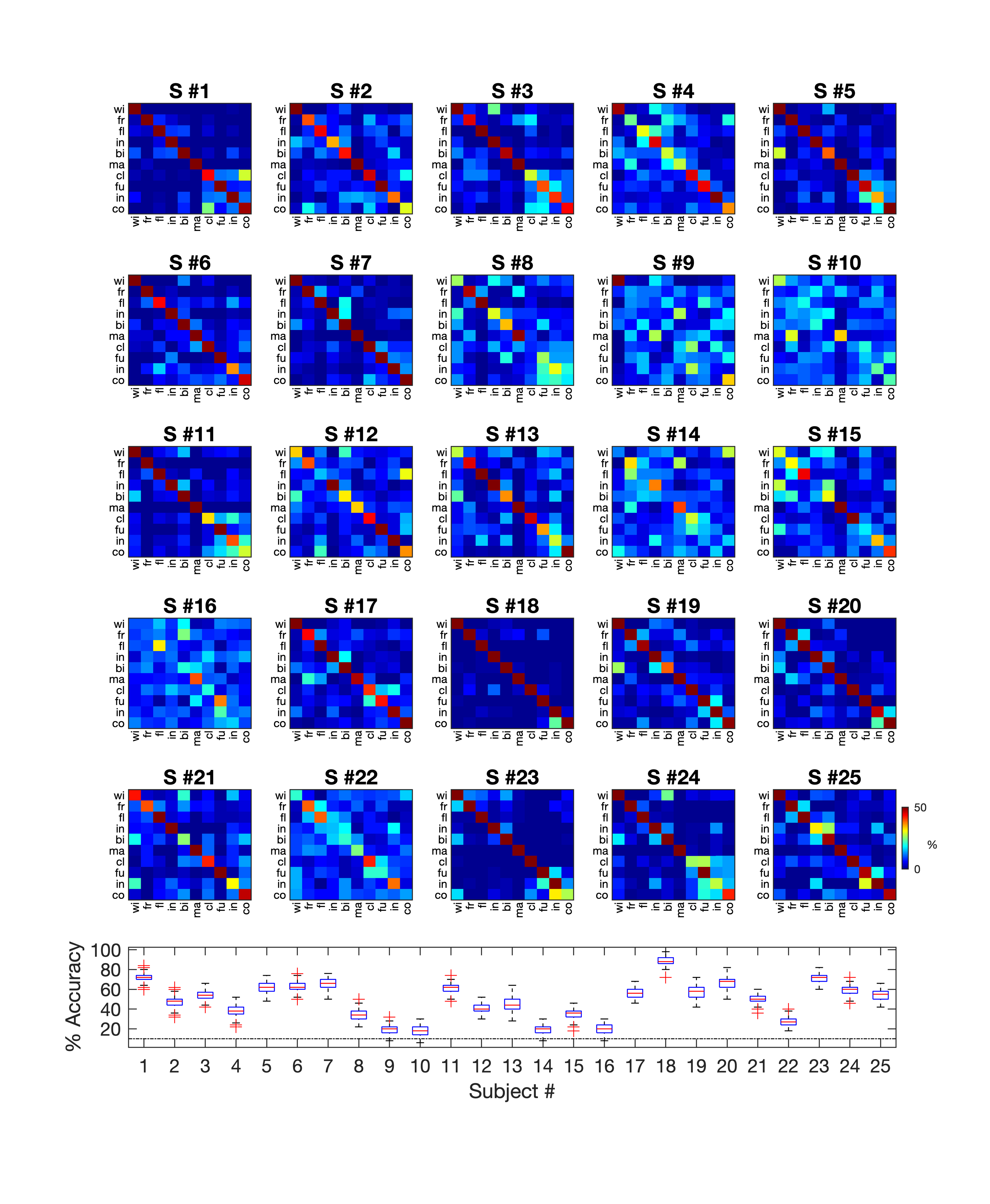

Supplement: S4 Fig — Analog to Fig 4. Depicted are confusion matrices of decoding analyses based on data of each individual subject, collapsed across anatomical regions and sessions (see Decoding of stimulus identity and category section in Materials and methods). Decoders were trained to predict the category label of stimuli, trained on data of half of the stimuli in each category. Out-of-sample accuracies in 100 random subdivisions of data into training and test sets for each of the 25 subjects are depicted in the box plots beneath the confusion matrices. Note that boxes of decoding accuracies are above chance level (dotted line, 10%) in all subjects. Successful out-of-sample decoding on new exemplars of the category indicates an abstract semantic code implemented in the neuronal firing of MTL regions. Data and scripts underlying this figure are deposited here: https://github.com/rebrowski/abstractRepresentationsInMTL. MTL, medial temporal lobe. (TIF) [file pbio.3000290.s004.tif]

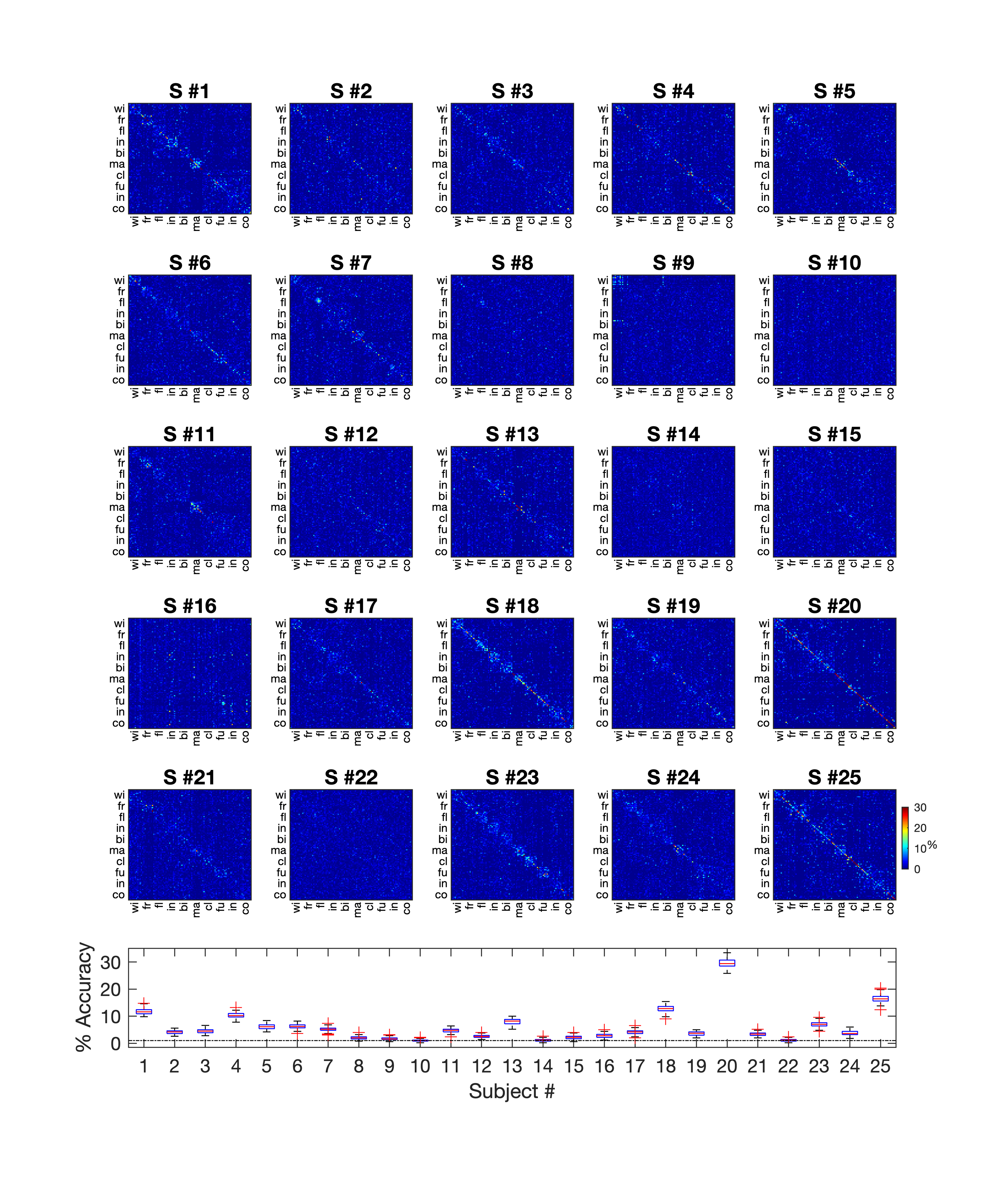

Supplement: S5 Fig — Analog to Fig 4. Depicted are confusion matrices of decoding analyses based on data of each individual subject, collapsed across anatomical regions and sessions (see Decoding of stimulus identity and category section in Materials and methods). Decoders were trained to predict the label of stimuli and trained on data of half of the trials per stimulus. Out-of-sample accuracies in 100 random subdivisions of data into training and test sets for each subject are depicted in the box plots beneath the confusion matrices. Note that boxes of decoding accuracies are above chance (dotted line, 1%) in all subjects and that confusions between stimuli occur more often within rather than across category. Data and scripts underlying this figure are deposited here: https://github.com/rebrowski/abstractRepresentationsInMTL. (TIF) [file pbio.3000290.s005.tif]
